# Palliative Care Service Use in Four European Countries: A Cross-National Retrospective Study via Representative Networks of General Practitioners

**DOI:** 10.1371/journal.pone.0084440

**Published:** 2013-12-30

**Authors:** Lara Pivodic, Koen Pardon, Lieve Van den Block, Viviane Van Casteren, Guido Miccinesi, Gé A. Donker, Tomás Vega Alonso, José Lozano Alonso, Pierangelo Lora Aprile, Bregje D. Onwuteaka-Philipsen, Luc Deliens, on behalf of EURO IMPACT

**Affiliations:** 1 End-of-Life Care Research Group, Vrije Universiteit Brussel (VUB) and Ghent University, Brussels, Belgium; 2 Scientific Institute of Public Health (Wetenschappelijk Instituut Volksgezondheid, Institut Scientifique de Santé Publique), Unit of Health Services Research, Brussels, Belgium; 3 Clinical and Descriptive Epidemiology Unit, Cancer Prevention and Research Institute, Florence, Italy; 4 Dutch Sentinel General Practice Network, NIVEL, Netherlands Institute for Health Services Research, Utrecht, The Netherlands; 5 Public Health General Directorate, Regional Ministry of Health (Dirección General de Salud Pública, Consejería de Sanidad), Castile and Leon, Valladolid, Spain; 6 Italian Society of General Medicine (Società Italiana di Medicina Generale), Florence, Italy; 7 Department of Public and Occupational Health, EMGO+ Institute, VU University Medical Center, Amsterdam, The Netherlands; University of St Andrews, United Kingdom

## Abstract

**Background:**

Due to a rising number of deaths from cancer and other chronic diseases a growing number of people experience complex symptoms and require palliative care towards the end of life. However, population-based data on the number of people receiving palliative care in Europe are scarce. The objective of this study is to examine, in four European countries, the number of people receiving palliative care in the last three months of life and the factors associated with receiving palliative care.

**Methods:**

Cross-national retrospective study. Over two years (2009–2010), GPs belonging to representative epidemiological surveillance networks in Belgium, the Netherlands, Italy, and Spain registered weekly all deaths of patients (≥18 years) in their practices and the care they received in the last three months of life using a standardized form. Sudden deaths were excluded.

**Results:**

We studied 4,466 deaths. GPs perceived to have delivered palliative care to 50% of patients in Belgium, 55% in Italy, 62% in the Netherlands, and 65% in Spain (p<.001). Palliative care specialists attended to 29% of patients in the Netherlands, 39% in Italy, 45% in Spain, and 47% in Belgium (p<.001). Specialist palliative care lasted a median (inter-quartile range) of 15 (23) days in Belgium to 30 (70) days in Italy (p<.001). Cancer patients were more likely than non-cancer patients to receive palliative care in all countries as were younger patients in Italy and Spain with regard to specialist palliative care.

**Conclusions:**

Although palliative care is established in the countries studied, there are considerable differences in its provision. Two potentially underserved groups emerge non-cancer patients in all countries and older people in Italy and Spain. Future research should examine how differences in palliative care use relate to both patient characteristics and existing national health care policies.

## Introduction

It is estimated that, in Europe, 4.8 million people die every year. Approximately two million die from serious chronic disease and cancer, with a further increase in deaths from these causes expected [1]. Given that a growing proportion of people will live into old and very old age, and that chronic diseases are more common in old people, an increasing number of people will be living with the effects of these illnesses [2], [3]. The burden that these demographic and epidemiological developments place on society has been recognized by the World Health Organization (WHO) and the European Union who have identified care for people at the end of life as an important public health issue [4], [5].

People dying from cancer and other serious chronic diseases are very likely to experience multiple and complex symptoms requiring appropriate and timely assessment and treatment, and thus are in potential need of palliative care [2]. The WHO defines palliative care as “…an approach that improves the quality of life of patients and their families facing the problem associated with life-threatening illness, through the prevention and relief of suffering by means of early identification and impeccable assessment and treatment of pain and other problems, physical, psychosocial and spiritual.” [4] Population-based data on the number and characteristics of people who access palliative care in Europe are limited. Particularly little is known about the characteristics of people who do not receive palliative care, and about the extent to which palliative care is delivered to those dying from illnesses other than cancer, who are likely to be underserved [6], [7]. Obtaining this information is essential for ensuring the adequate organisation and provision of palliative care on national levels, and its integration into mainstream health care services.

In recent years major efforts have been undertaken to assess the establishment of palliative care within health care systems around the world, including the availability of palliative care services, funding arrangements and policy support [8]–[11]. While these studies provide valuable knowledge about the development of palliative care, they do not provide information on the actual percentage of people at the end of life receiving it nor about the timing of initiation of palliative care before death. Other studies have focussed on single countries only or have selectively studied patients with cancer or those in particular care settings, thereby precluding a population-based perspective on the use of palliative care [12]–[15]. Studies estimating population coverage solely on the basis of annual activity data of palliative care services risk double-counting, since unique patient codes are not used across health care settings. Additionally, due to differences in research design, these single studies cannot be compared across countries.

We have conducted one of the first population-based studies assessing and comparing the frequency and timing of use of palliative care in a representative sample of the general population of patients at the end of life in four European countries. Earlier studies in Belgium and the Netherlands have successfully applied a similar design, but did not explicitly assess whether GPs delivered palliative care nor the length of time over which patients received palliative care [16]–[18]. We have extended the study design to include two more countries, Italy and Spain, and have assessed the delivery of palliative care by GPs and by specialist palliative care services as well as the time of initiation.

This study aimed to address the following research questions with regard to the last three months of life of patients who died non-suddenly in Belgium, the Netherlands, Italy and Spain:

How many people receive palliative care from their GP and through specialist palliative care services, and are there differences between countries?

For how many days during the last three months of life do people receive specialist palliative care, and are there differences between countries?

Are patients’ sex, age, cause and place of death associated with the use of palliative care delivered by the GP and the use and time of initiation of specialist palliative care?

## Methods

### Ethics Statement

#### Informed consent and patient and GP anonymity

The participating GPs gave written informed consent at the beginning of each registration year, after being fully informed about the objectives and methods of the study. Strict procedures regarding patient anonymity were followed during data collection and entry. Each registered death received an anonymous reference from the GP. Any potentially identifying patient and GP data (such as patient’s date of birth, postcode, and GP identification number) were replaced by aggregate categories or anonymous codes. In Belgium and the Netherlands, patients of GPs who were part of sentinel networks were informed through posters or leaflets displayed in the practices that their data could be used anonymously for research purposes. In Italy, patients are informed that their care-related data, when anonymised, can be used to monitor care as standard. Patients in Spain were not necessarily informed that their data could be used for research. However, as all data were thoroughly anonymised, this was not required. The proceedings in each country comply with the country’s respective laws [19]–[25].

#### Ethics approval

Ethics approval for this study, including the consent procedure, was obtained from the Ethical Review Board of Brussels University Hospital of the Vrije Universiteit Brussel, Belgium (2004), and from the Local Ethical Committee ‘Comitato Etico della Azienda U.S.L. n. 9 di Grosseto’ in Tuscany, Italy (2008). Ethics approval was not required for posthumous collection of anonymous patient data in the Netherlands [19], [20] or Spain [21]–[23], according to the legislation of these countries.

### Study Design

This study is part of the EURO SENTIMELC (European Sentinel Network Monitoring End-of-Life Care) study, a cross-national retrospective study monitoring end-of-life care in Belgium, the Netherlands, Italy, and Spain (the Castile and Leon and Valencian Community regions) [26]. Data were collected through representative sentinel networks of GPs who continuously registered all deaths amongst patients in their practices. GP sentinel networks are epidemiological surveillance networks consisting of freestanding practices or community-based physicians who voluntarily and continuously monitor health problems occurring in the population. They have a long-standing involvement in epidemiological research, a low annual turnover, and were shown to be suitable for monitoring health-related epidemiological data [27]–[29]. In Belgium, the Netherlands, and Spain we cooperated with existing long-standing sentinel networks that monitor a wide range of health problems, whereas in Italy a new network was formed for this study as the existing network had a flu surveillance focus. GPs are recruited into the sentinel networks by national public health institutes, who draw a random sample of GPs and invite them to become part of the network. The new Italian network was created by the Italian Society of General Practitioners through a procedure similar to that in the other countries. The Italian GPs were recruited from nine health districts that are spread all over the country. At the point of recruitment, GPs were informed only about the procedure and not about the subject of the surveillance in order to avoid an overrepresentation of GPs with a particular interest in palliative care. In each country the institutes responsible for creating the sentinel networks verified that the sentinel GPs were representative of the total GP population in terms of age, sex, and geographic location. The number of participating GPs and their population coverage per country in 2009 was as follows: 199 (1.8%) in Belgium, 59 (0.8%) in the Netherlands, and 149 (4.3%) in Italy. In Spain, data were collected in 2010 only. The respective figures for 2010 are 189 (1.5%) in Belgium, 63 (0.8%) in the Netherlands, 94 (2.7%) in Italy, and 173 in Spain (114 (3.8%) in Castile and Leon, 59 (2.2%) in the Valencian Community). The percentages in Italy refer to the population of the nine participating health districts. The percentages in Spain refer to the population aged 18 years or over. The participating GPs of all four countries had an adequate geographical distribution and were representative of the general population of GPs in the respective country (or region in Spain) with regard to sex and age [26]. This study protocol has been successfully implemented in a number of cross-national comparisons [17], [30]–[32]. Further information on the sentinel networks of GPs and the methods of the study is provided in the published study protocol [26].

### Sample

Deaths of all patients 18 years or older in the participating GP practices were included. Deaths classified by the GP as sudden and totally unexpected, and those for which this information was missing, were excluded in order to obtain a sample of patients for whom palliative care was a relevant consideration [33], [34]. This criterion was chosen following a careful consideration of its methodological strengths and limitations [34], and was based on the fact that several studies in the palliative care literature distinguish between sudden and non-sudden deaths [33]–[36]. Furthermore, nursing home deaths in the Netherlands were excluded, as these patients are not cared for by GPs but by specialised nursing home physicians.

### Procedure

Using a standardised form GPs belonging to the sentinel networks registered weekly all deaths among patients in their practice who were aged 18 years or over throughout a two year period, from Jaunary 1^st^ 2009 to December 31^st^ 2010 (in Spain over one year, in 2010). At the beginning of each year, GPs received instructions, clearly stating the inclusion criteria of the study and how to complete the registration form.

### Measurement

The registration form consists of structured and closed-ended items surveying care-related information about the patient’s final three months of life. The literature suggests that this is a relevant time period for studying end-of-life care [33], [37], [38]. The registration form was initially developed in Dutch and French and translated into English. From English it was subsequently translated into Italian and Spanish. All translations were carried out via forward-backward procedure. The following items of the registration form were included in the present study:

Dependent variables:

The GPs’ perception of whether she or he had delivered palliative care to the patient : ‘did you provide palliative care to this patient?’.

Specialist palliative care: ‘which palliative care initiatives were involved in the last 3 months of this patient’s life?’ GPs were asked to indicate which palliative care initiatives from a list of options were involved in the patient’s care (these options differed between countries due to differences in the services that are available, see Table 1). The terminology used to label these services corresponds to the labels commonly used in primary care in the respective countries.

**Table 1:** Classification of specialist palliative care services and healthcare professionals involved in these services.

Category	Belgium	Netherlands	Italy	Spain
**Hospice/palliative care unit**	Palliative care unit in a hospital: physician and nurses; can call on the support of other professionals (psychologists, social workers, etc.)	Hospice, palliative care unit (in a hospital, nursing home, or care home): palliative care nurses, volunteers, patient’s GP, sometimes hospice doctor or other medical specialist	Hospice: physicians and nurses specialised in palliative care, can call on the support of other professionals (psychologists, social workers, etc.)	Palliative care unit in a hospital: physicians and nurses specialised in palliative care, psychologist
**Palliative care service for patients staying at home**	Palliative home care team: one GP, two nurses, one secretarial assistant. Their aim it is to advise front-line carers.Palliative day care centre: physician, palliative care nurses, volunteers	Palliative care consultation team: consult all professionals involved in palliative care; consist of palliative care nurses, GPs, nursing home physicians, and other medical specialists, all with extra training or expertise in palliative care*	Palliative home care team: physician and nurse specialised in palliative care; limited contact with GPs. Domiciliary integrated assistance with palliative care: GP and nurse	Palliative home care team: GP, nurse, social worker, psychologist. Palliative day care centre: GP and nurse. Ambulatory palliative care in a hospital: physicians and nurses specialised in palliative care
**GP with formal palliative care training**	§	GP with palliative care training†	§	§
**In-house palliative care service in a nursing home**	Reference persons for palliative care in a nursing home: aim to support GPs and nurses operating in the nursing home	§	§	Palliative care nurses in a nursing home
**Hospital-based palliative care service (excl. palliative care unit)** ‡	Mobile palliative care support team in a hospital: mobile team operating within the hospital, consisting of specially trained staff physicians, nurses, and paramedics	Palliative care consultation team (see above)*	Pain therapy or palliative care specialist consultation during a hospital admission: physicians specialised in palliative care	§

Palliative care consultation teams offer services to patients at home as well as to patients in hospital/hospice/nursing home. Seventy-seven per cent of those for whom palliative care consultation is requested are cared for at home [43].

GPs who followed a ‘training in palliative care for general practitioners with an advisory role’ offered by the Dutch Association of General Practitioners (Nederlands Huisartsen Genootschap, NHG) and who are registered as palliative care advisors in a central database.

For patients admitted to hospital for at least one day in the last three months of life.

No specialist palliative care initiatives available.

Time of initiation of specialist palliative care: ‘estimate the number of days between the first palliative intervention and the moment of death’.

Independent variables:

Age at death and sex.

Cause of death: ‘illness or disorder that was the direct cause of death’.

Place of death: (a) home/living with family, (b) care home/home for the elderly/nursing home, (c) hospital (excl. palliative care unit and nursing home unit in a hospital), (d) palliative care unit/hospice, (e) elsewhere (specified).

As the types of specialist palliative care services differ between countries, they were classified into five categories to facilitate comparison (Table 1).

While the geographic distribution of palliative care services is relatively homogenous in Belgium and the Netherlands, there is large heterogeneity in Italy and Spain. In Italy, the density of palliative care services is higher in the north of the country compared to the south, and in Spain there is great variation in the organisation of palliative care between the different Autonomous Communities. Furthermore, the countries differ with regard to the role of GPs within palliative care services. In the Netherlands and Belgium GPs are seen as the main care providers for people requiring palliative care [39], [40] and in Spain they are core providers of palliative care in the community [41]. However, there is uncertainty about the role of GPs within specialist palliative care teams in Italy, perhaps due to the heterogeneity of organizational models in the country [42].

### Statistical Analysis

We carried out χ^2^-tests to analyse differences in sex, age, cause and place of death between the samples of the countries studied. The place of death category ‘other’ was not included in the significance tests in case of cell frequencies below 5.

Binary multivariate logistic regression analyses, adjusted for age, sex, cause and place of death, were computed to assess the association between country as the independent variable and receiving palliative care by the GP and specialist palliative care as dependent variables. A further binary multivariate logistic regression analysis, adjusted for age, sex, cause and place of death, was computed to compare between countries the number of patients attended to by different specialist palliative care services. The number of days during which specialist palliative care was provided in the last three months of life was analysed both as a continuous and as a categorical variable. The categories were (1) 1–3 days, (2) 4–7 days, (3) 8–30 days, and (4) 31–92 days. The continuous variable was compared between countries using the Kruskal-Wallis test. A multivariate ordinal logistic regression analysis, adjusted for age, sex, cause and place of death, was conducted to test the association between country and the four categories of time of initiation.

For each country two binary multivariate logistic regression analyses were computed to test whether age, sex, cause and place of death are associated with receiving palliative care by the GP (first analysis) or specialist palliative care services (second analysis). Finally, a multivariate ordinal logistic regression analysis was computed for each country to test whether the same factors are associated with time of initiation of specialist palliative care.

For each multivariate logistic regression analysis commonly recommended assumptions were tested [44]. Odds ratios and binomial 95% confidence intervals were calculated for all dependent variables and p-values for all tests of significance. All statistical tests were performed with a significance level of α <0.05 in IBM SPSS Statistics (version 19). If the percentage of missing values was below 10%, cases with missing values on a particular variable were omitted in analyses involving the respective variable. In case it amounted to more than 10% we tested whether the proportion of missing values was significantly associated with patients having received palliative care.

## Results

### Sample Characteristics

In total, 6,858 deaths were reported, of which 4,518 (65.9%) were classified by the GPs as expected or non-sudden. The percentage of deaths judged as non-sudden per country was as follows: 67% in Belgium, 62% in the Netherlands, 66% in Italy, 69% in Spain (p<.001). These percentages of non-sudden deaths are in line with those reported by physicians in previous studies with a similar methodology [33]–[36]. Following the exclusion of nursing home deaths from the Netherlands (n = 52, 7.6% of all deaths registered in the Netherlands) a sample of 4,466 deaths remained of which 1,604 were registered in Belgium, 635 in the Netherlands, 1,839 in Italy, and 388 in Spain. The samples of the registered non-sudden deaths from Belgium and the Netherlands (excluding nursing home deaths) were compared to those of previous death certificate studies in which representative samples of non-sudden deaths were obtained [38], [45]. For Italy and Spain, the sample of all registered deaths was compared with national mortality statistics. We did not find large differences, except for a slight underrepresentation of non-sudden hospital deaths and people under the age of 65 in Belgium and women in the Netherlands [26].

Differences between countries in terms of sex, age, cause and place of death were statistically significant (Table 2). The Dutch sample revealed the smallest percentage of people aged 85 years or over and the highest percentage of cancer deaths. In Spain there was a relatively larger number of men and lower number of women compared with the other countries. In Belgium, most people died in hospital, whereas home was the most frequent place of death in the other countries.

**Table 2:** Characteristics of non-sudden deaths in Belgium, the Netherlands, Italy and Spain; % (95% CI), n.

Patient characteristics		Belgium	Netherlands	Italy	Spain	p-value
		N = 1604	N = 635	N = 1839	N = 388	
Sex						.04*
	Male	46 (44 to 48)	47 (43 to 51)	47 (45 to 49)	54 (49 to 59)	
		731	295	856	209	
	Female	54 (52 to 56)	53 (49 to 57)	53 (51 to 55)	46 (41 to 51)	
		868	333	983	179	
Age at death						<.001*
	18–64 y	14 (12 to 16)	18 (15 to 21)	13 (11 to 15)	11 (8 to 14)	
		219	117	233	43	
	65–84 y	47 (45 to 49)	50 (46 to 54)	47 (45 to 49)	45 (40 to 50)	
		753	318	860	174	
	≥85 y	39 (37 to 41)	32 (28 to 36)	40 (38 to 42)	44 (39 to 49)	
		620	200	746	171	
Cause of death						<.001*
	Cancer	37 (35 to 39)	53 (49 to 57)	46 (44 to 48)	39 (34 to 44)	
		595	335	830	149	
	Cardiovasculardiseases	15 (13 to 17)	15 (12 to 18)	21 (19 to 23)	16 (12 to 20)	
		237	94	375	63	
	Respiratory disease	11 (9 to 13)	8 (6 to 10)	7 (6 to 8)	14 (11 to 17)	
		171	49	130	55	
	Diseases of the nervous system	7 (6 to 8)	3 (2 to 4)	6 (5 to 7)	5 (3 to 7)	
		114	19	105	18	
	Stroke	7 (6 to 8)	4 (2 to 6)	10 (9 to 11)	11 (8 to 14)	
		109	24	181	41	
	Other	23 (21 to 25)	18 (15 to 21)	10 (9 to 11)	15 (11 to 19)	
		376	112	173	58	
Place of death						<.001*
	Home	23 (21 to 25)	44 (40 to 48)	46 (44 to 48)	49 (44 to 54)	
		367	276	846	188	
	Care home	31 (29 to 33)	18 (15 to 21)	9 (8 to 10)	13 (10 to 16)	
		499	114	164	48	
	Hospital	36 (34 to 38)	28 (25 to 31)	39 (37 to 41)	33 (28 to 38)	
		577	177	716	128	
	Palliative care unit/hospice	9 (8 to 10)	10 (8 to 12)	5 (4 to 6)	4 (2 to 6)	
		150	66	101	17	
	Other†	0	0	1 (0.5 to 1.5)	1 (0.005 to 2)	
		4	1	9	3	

CI = confidence interval.

Percentages are within-country percentages. Percentages are rounded and thus may not add up to 100.

Nursing home deaths from the Netherlands (n = 52) were excluded.

Missing values: age n = 12 (0.3%), sex n = 12 (0.3%), place of death n = 15 (0.3%), cause of death n = 53 (1.2%).

Pearson *χ* ^2^ test.

Not included in significance tests.

### Palliative Care Provision by GPs

GPs stated they had delivered palliative care themselves to 50% (Belgium), 55% (Italy), 62% (Netherlands), and 65% (Spain) of patients (p<.001) (Table 3). GPs in Spain indicated that they had delivered palliative care to significantly more patients than GPs in Italy and Belgium. No statistically significant difference emerged between GPs’ reports in Spain and the Netherlands.

**Table 3:** Use of palliative care provided GPs and use of and number of days in specialist palliative care in the last three months of life; % (95% CI), n.

		Belgium	Netherlands	Italy	Spain	p-value†
		N = 1604	N = 635	N = 1839	N = 388	
Received specialist palliative care*		47 (44 to 49)	29 (25 to 33)	39 (37 to 41)	45 (40 to 50)	<.001
		717	172	683	174	
Received palliative care by GP according to GP’s self-report*		50 (48 to 53)	62 (58 to 65)	55 (53 to 57)	65 (60 to 70)	<.001
		807	375	1005	239	
In case specialist palliative care was provided		N = 717	N = 172	N = 683	N = 174	p-value
Time of initiation of specialist palliative care						
	Median (IQR)	15 (23)	21 (53)	30 (70)	26 (53)	<.001‡
	1–3 days	11 (9 to 13)	9 (4 to 13)	3 (2 to 4)	8 (3 to 13)	<.001
		78	14	17	11	
	4–7 days	18 (15 to 21)	18 (12 to 24)	6 (4 to 8)	17 (11 to 23)	
		127	29	36	23	
	8–30 days	47 (43 to 51)	38 (31 to 46)	42 (38 to 46)	36 (28 to 45)	
		330	62	240	50	
	31–92 days	24 (20 to 27)	35 (28 to 43)	49 (45 to 53)	39 (31 to 47)	
		165	57	278	53	

IQR = inter-quartile range; CI = confidence interval.

Percentages are within-country percentages. Percentages are rounded and thus may not add up to 100.

Missing values: SPC n = 191 (4.3%); time of initiation of SPC n = 174 (3.9% of those who received SPC); palliative care by GP n = 55 (1.2%).

Palliative care categories are not mutually exclusive.

p-values based on multivariate analyses adjusted for age, sex, cause and place of death.

Kruskal-Wallis test (bivariate analysis).

### Use and Duration of Specialist Palliative Care Services

Varying by country, 47% (Belgium), 29% (Netherlands), 39% (Italy), and 45% (Spain) of patients received specialist palliative care in the last three months of life (p<.001) (Table 3). This percentage was significantly higher in Belgium, Spain, and Italy compared to the Netherlands. The median number of days (and inter-quartile range) over which specialist palliative care was received in the last three months of life was 15 (23), 21 (53), 26 (53), and 30 (70) in Belgium, the Netherlands, Spain, and Italy, respectively (p<.001). The four countries differed significantly with regards to the percentage of patients cared for in the different specialist palliative care settings (Table 4). In Italy and Spain specialist palliative care was mostly delivered through services for patients residing at home (for 24% and 29% of patients, respectively), in Belgium for patients residing at home and in a nursing home (16% of patients in each setting) and in the Netherlands for those residing in a hospice or palliative care unit (14%) or receiving care from a GP with palliative care training (12%). The lowest proportion of patients to receive hospital-based palliative care services, not including those in palliative care units, was found in the Netherlands (1%).

**Table 4:** Use of specialist palliative care services by country; % (95% CI), n.

Specialist palliative care service*	Belgium	Netherlands	Italy	Spain	p-value†
	N = 1604	N = 635	N = 1839	N = 388	
Hospice/palliative care unit	11 (10 to 13)	14 (11 to 17)	8 (6 to 9)	17 (13 to 21)	<.001
	174	83	132	66	
Palliative care service for patientsstaying at home	16 (14 to 18)	5 (4 to 7)	24 (22 to 26)	29 (24 to 33)	<.001
	250	32‖	418	111	
GP with formal palliative care training		12 (10 to 15)			¶
	§	74	§	§	
In-house palliative care service in anursing home	16 (14 to 17)			5 (1 to 8)	<.07‡
	239	§	§	21	
Hospital-based palliative care service (excl. palliative care unit)	12 (10 to 13)	1 (0.4 to 2)	11 (10 to 13)		<.001
	179	8‖	196	§	

CI = confidence interval.

Percentages are within-country percentages. Percentages are rounded and thus may not add up to 100.

Missing values: specialist palliative care n = 191 (4.3%).

Palliative care categories are not mutually exclusive.

p-values are based on multivariate analyses adjusted for age, sex, cause and place of death.

Statistically significant in bivariate analysis.

Palliative care initiative not present in this country.

Palliative care consultation teams in the Netherlands provide services to people at home and in hospital. Our data do not hold information as to where the patients received this service.

Comparison between countries not possible.

### Factors Associated with Specialist Palliative Care and with GPs’ Self-reports of Palliative Care Provision

Results of the multivariate logistic regression analyses indicate that in all countries, except the Netherlands, a cancer diagnosis was a significant predictor for receiving specialist palliative care and for GPs indicating that they provided palliative care to the patient (Table 5). In the Netherlands this applied only to GPs’ reports of palliative care provision. In Italy and Spain, being younger than 85 years was associated with higher chances of receiving specialist palliative care and, in addition, in Italy being younger than 65 years resulted in lower chances of GPs reporting that they provided palliative care. Age was not significantly related to the provision of palliative care by either GPs or palliative care specialists in Belgium and the Netherlands. According to the GPs’ report, people who died at home were more likely to have received palliative care by the GP than people who died in other locations (except for hospice/palliative care unit in Italy and Spain). Compared with dying at home, dying in hospital was associated with a lower chance of receiving specialist palliative care in Belgium and the Netherlands, but not in Italy and Spain.

**Table 5:** Factors associated with use of palliative care provided by GPs and specialist palliative care services*.

			GP palliative care†		Specialist palliative care†	
Patient and health care characteristics			Received % (n)	OR (95% CI)	Received % (n)	OR (95% CI)
Belgium‖‖			N = 1573			N = 1512
Age				p = .872		p = .397
	≥85 y		54 (329)	Ref	42 (251)	Ref
	65–84 y		47 (349)	0.96 (0.73 to 1.27)	47 (339)	1.16 (0.89 to 1.51)
	18–64 y		52 (112)	0.90 (0.59 to 1.36)	56 (114)	1.28 (0.85 to 1.92)
Sex				p = .435		p = .297
	Male		46 (331)	Ref	45 (307)	Ref
	Female		54 (459)	1.11 (0.86 to 1.43)	48 (397)	1.14 (0.89 to 1.46)
Cause of death				p<.001		p<.001
	Non-cancer		43 (424)	Ref	36 (342)	Ref
	Cancer		63 (366)	**4.60 (3.37 to 6.28)**	65 (362)	**2.93 (2.24 to 3.85)**
Place of death				p<.001		p<.001
	Home		77 (276)	Ref	48 (172)	Ref
	Care home		69 (338)	1.06 (0.75 to 1.48)	50 (242)	**1.72 (1.26 to 2.36)**
	Hospital		19 (108)	**0.07 (0.05 to 0.10)**	27 (142)	**0.48 (0.36 to 0.65)**
	Palliative care unit/hospice		46 (68)	**0.14 (0.09 to 0.23)**	100 (148)	§
	Other‡		0 (0)		0 (0)	
Netherlands‖‖			N = 599			N = 585
Age				p = .942		p = .383
	≥85 y		60 (110)	Ref	22 (40)	Ref
	65–84 y		62 (189)	0.98 (0.59 to 1.64)	32 (94)	1.50 (0.84 to 2.68)
	18–64 y		66 (72)	1.09 (0.54 to 2.20)	33 (35)	1.33 (0.62 to 2.83)
Sex				p = .064		p = .629
	Male		61 (176)	Ref	32 (86)	Ref
	Female		63 (195)	1.51 (0.98 to 2.33)	27 (83)	1.12 (0.71 to 1.78)
Cause of death				p<.001		p = .477
	Non-cancer		47 (129)	Ref	19 (51)	Ref
	Cancer		75 (242)	**2.44 (1.51 to 3.93)**	37 (118)	1.22 (0.71 to 2.08)
Place of death				p<.001		p = .001
	Home		87 (228)	Ref	27 (69)	Ref
	Care home		67 (71)	**0.40 (0.22 to 0.73)**	20 (22)	0.81 (0.44 to 1.49)
	Hospital		20 (33)	**0.04 (0.03 to 0.08)**	8 (13)	**0.25 (0.13 to 0.49)**
	Palliative care unit/hospice		61 (39)	**0.21 (0.11 to 0.39)**	100 (65)	§
	Other‡		0 (0)		0 (0)	
Italy‖‖			N = 1777			N = 1709
Age				p = .062		p = .001
	≥85 y		55 (389)	Ref	25 (176)	Ref
	65–84 y		56 (471)	0.93 (0.74 to 1.16)	45 (356)	**1.46 (1.12 to 1.89)**
	18–64 y		53 (119)	**0.67 (0.47 to 0.94)**	64 (140)	**2.02 (1.38 to 2.97)**
Sex				p = .433		p = .476
	Male		55 (459)	Ref	42 (341)	Ref
	Female		55 (520)	1.08 (0.89 to 1.32)	37 (331)	1.09 (0.86 to 1.37)
Cause of death				p<.001		p<.001
	Non-cancer		48 (462)	Ref	19 (174)	Ref
	Cancer		63 (517)	**1.95 (1.57 to 2.43)**	63 (498)	**5.19 (4.07 to 6.62)**
Place of death				p<.001		p = .473
	Home		63 (515)	Ref	35 (277)	Ref
	Care home		48 (75)	**0.59 (0.42 to 0.83)**	31 (45)	1.34 (0.88 to 2.04)
	Hospital		47 (324)	**0.53 (0.43 to 0.65)**	38 (249)	1.15 (0.90 to 1.46)
	Palliative care unit/hospice		64 (65)	0.85 (0.54 to 1.32)	100 (101)	§
	Other‡		0 (0)		0(0)	
			GP palliative care		Specialist palliative care	
Patient and health care characteristics			Received n (%)	OR (95% CI)	Received n (%)	OR (95% CI)
Spain‖‖			N = 357			N = 378
Age				p = .952		p<.001
	≥85 y		64 (98)	Ref	29 (47)	Ref
	65–84 y		66 (107)	1.06 (0.63 to 1.79)	54 (92)	**2.47 (1.49 to 4.12)**
	18–64 y		70 (28)	1.15 (0.47 to 2.82)	73 (30)	**5.52 (2.29 to 13.33)**
Sex				p = .363		p = .465
	Male		63 (123)	Ref	47 (95)	Ref
	Female		68 (110)	1.25 (0.78 to 2.01)	43 (74)	1.19 (0.75 to 1.90)
Cause of death				p = .008		p = .004
	Non-cancer		59 (128)	Ref	34 (78)	Ref
	Cancer		76 (105)	**2.12 (1.22 to 3.70)**	62 (91)	**2.08 (1.26 to 3.44)**
Place of death				p<.001		p = .145
	Home		78 (139)	Ref	42 (79)	Ref
	Care home		52 (23)	**0.37 (0.18 to 0.75)**	47 (21)	1.80 (0.90 to 3.60)
	Hospital		51 (61)	**0.29 (0.17 to 0.49)**	41 (52)	0.75 (0.45 to 1.25)
	Palliative care unit/hospice		67 (10)	0.42 (0.13 to 1.36)	100 (17)	§
	Other‡		0 (0)		0 (0)	

OR = odds ratio; CI = confidence interval; Ref = reference category.

All percentages indicate proportions within the independent variable. Percentages are rounded and thus may not add up to 100.

Missing values for dependent variables: specialist palliative care n = 191 (4.3%); GP palliative care n = 55 (1.2%); missing values for independent variables: age n = 12 (0.3%), sex n = 12 (0.3%), cause of death n = 53 (1.2%), place of death n = 15 (0.3%).

Odds ratios in bold indicate statistically significant associations.

Independent variables age and cause of death were correlated (r = .40, p<.01). Variance inflation factors did not indicate problems of multicollinearity.

Two multivariate logistic regression analyses with 1) palliative care by the GP and 2) specialist palliative care as dependent variable.

Specialist palliative care and palliative care by the GP are not mutually exclusive categories.

Not included in significance tests.

OR not meaningful as 100% of cases have the same value on the dependent variable.

Missing values on the independent variables resulted in missing cases in the multivariate logistic regression analyses. The number of deaths included in the analyses are indicated.

### Factors Associated with Time of Initiation of Specialist Palliative Care

Ordinal logistic regression analyses revealed that a cancer diagnosis, compared with a non-cancer diagnosis, was related to higher chances of an early initiation of specialist palliative care in Belgium (OR = 1.79 [1.27 to 2.53]) and the Netherlands (OR = 4.21 [1.90 to 9.30]), but not in Italy and Spain. Dying in a palliative care unit (OR = 0.59 [0.39 to 0.91]) or a hospital (OR = 0.44 [0.29 to 0.68]) in Belgium or in a palliative care unit in Spain (OR = 0.27 [0.07 to 0.97]) was associated with a later initiation of specialist palliative care compared with dying at home. Time of initiation of palliative care was not significantly related to either age or sex in any of the countries, nor to place of death in the Netherlands and Italy.

## Discussion

Of the deaths registered for this study, GPs judged 66% as non-sudden and expected. This percentage of non-sudden deaths is consistent with percentages reported by physicians in earlier studies in six European countries [33]–[36] but lower than an estimate obtained in Australia that was based on cause of death and amounted to 89% of all deaths [46]. The percentage of non-sudden deaths differed significantly between countries. This was very likely caused by the exclusion of nursing home deaths from the Netherlands.

Between half (Belgium) and two-thirds (Spain) of GPs reported that they had delivered palliative care themselves. According to GPs’ reports, specialist palliative care was provided to 47% of patients who died non-suddenly in Belgium, 45% in Spain, 39% in Italy, and 29% in the Netherlands. GPs in Italy reported the longest duration of specialist palliative care over the last three months of life (median 30 days) and GPs in Belgium the shortest (median 15 days). In the Netherlands and Spain this was 21 and 26 days respectively. In all countries, dying from cancer as opposed to non-malignant disease emerged as a significant predictor for receiving specialist palliative care and for the GPs’ self-reports of palliative care provision, as did being below 85 years of age for receiving specialist palliative care in Italy and Spain.

As this study was conducted through sentinel networks of GPs it is important to note that GPs have somewhat different roles in the health care systems of each of the countries studied. GPs act as gatekeepers to specialised care in the Netherlands and Spain but they do not have this role in Belgium and are only partial gatekeepers in Italy. Despite not being gatekeepers in Belgium, GPs have an important role in the health care system, and 95% of the Belgian population have a GP whom they consult regularly [47]. In all four countries, the vast majority of the population regularly consult a GP [47]–[50].

This is one of the first cross-national, population-based studies to describe and compare the use of palliative care delivered by GPs and the initiation and use of specialist palliative care among people who died non-suddenly in four European countries. Through representative GP networks we obtained, with the exception of Dutch nursing home deaths, a representative sample of deaths irrespective of disease, treatment or place of residence. Taking all non-sudden deaths as the denominator and focusing on the last three months of life enabled us to study care at the end of life of those who did and did not receive palliative care and to focus on care that was actually delivered in the context of dying [34], [37].

This study also has limitations. Firstly, although the obtained percentage of non-sudden deaths is comparable to other studies in Europe [33]–[36], inaccuracies in the GPs’ judgment of deaths as sudden and totally unexpected cannot be fully excluded. Secondly, it was not possible to validate the information provided by the sentinel GPs for each registered death against an external criterion (e.g., hospital registries, insurance data, palliative care registries) due to the anonymous coding of deaths by the GP networks. However, the characteristics of the sentinel networks and their registration procedures, i.e. long-standing experience, weekly registrations, GPs trained in data collection, consistency checks of data, support the completeness and accuracy of their reports. Furthermore, the strength of using sentinel networks of GPs as observational units is to obtain information not collected in other databases nor the GPs’ medical files. Thirdly, our data did not permit us to examine the validity of the GPs’ self-report of palliative care provision. We could not determine whether the GPs’ personal definitions of palliative care are consistent with expert definitions and comparable between countries. It may be that some GPs label care for a person with a chronic life limiting illness palliative care whereas others may refer to it as usual GP care. The results of this study therefore reflect the delivery of what GPs perceive to be palliative care. Fourthly, due to the retrospective nature of the data collection, recall bias cannot be excluded. However, we attempted to limit this by instructing GPs to register deaths on a weekly basis. Finally, we did not have access to deaths in nursing homes in the Netherlands where a large proportion of very old people and people with dementia reside [51].

Research on palliative care needs has suggested that, given an ideal state of affairs, every person who dies non-suddenly should receive palliative care [46]. The results of our study suggest that varying proportions of people who need palliative care receive it during the final three months of life in the countries studied. While our data do not hold information about how many of those who died non-suddenly without receiving palliative care had actually needed it, there is evidence that a lack of palliative care service uptake equates with unmet need, particularly in non-cancer patients [52]. With regards to our study, this implies that particular attention should be paid to non-cancer patients who died without palliative care as they are particularly likely to have unmet needs.

Differences in palliative care provision are likely to reflect variations in the types of services available as well as the organisation of palliative care within the respective health care systems. Looking at specialist palliative care services, the findings of this study show a particularly high involvement in Belgium and Spain and a relatively low one in the Netherlands. The fact that Belgium has the highest ratio of specific palliative care resources per million inhabitants of the four countries studied might have contributed to this result [8], [11]. Another possible explanation lies in the relatively high number of hospital deaths in Belgium relative to the other countries. According to Belgian law, every hospital is obliged to have a specialist palliative care support team. In contrast, in the Netherlands where involvement of specialist palliative care is lowest among the countries studied, a strong emphasis is put on GPs as being the principal providers of formal care for terminally ill patients [39], [53]. However, the low percentage in the Netherlands might also partly result from the exclusion of nursing home deaths.

In the Netherlands and Spain, more GPs reported that they had provided palliative care compared to Belgium and Italy, possibly as a result of GPs being gatekeepers to more specialised health care in these countries. By being the first health care professional to be consulted by terminally ill patients, GPs may more frequently deliver palliative care themselves. However, the reason may also be that the GPs of these two countries are more likely to perceive the care they provide as palliative care whereas GPs in Italy and Belgium may view it as usual GP care. This does not mean that GPs do not have a key role in palliative care in the other two countries. In Belgium, GPs typically work together with specialist palliative care services that mainly have an advisory and supportive role [40]. In Italy GPs typically refer patients to integrated domiciliary assistance, a home care service managed by themselves and involving nurses and specialised physicians [49].

The number of patients who received palliative care does not permit conclusions about the appropriateness of care. The time of initiation before death, however, may be an indicator of adequacy as a late referral is often seen as a barrier to achieving the goals of palliative care [40]. Specialist palliative care appears to start latest in Belgium which might be a consequence of a Belgian regulation requiring a life expectancy of between 24 hours and three months in order for patients receiving palliative care at home to be granted a ‘palliative lump sum’ from their health insurance, to cover the costs of medicines, aids, and medical care materials. According to GPs’ reports, there is a relatively early initiation of specialist palliative care in Italy and Spain, on the other hand which could be influenced by the fact that no time limit is set by national health services for access to palliative care services. However, this result is somewhat surprising in light of literature suggesting insufficient communication between doctors and patients concerning diagnosis and prognosis in these two countries [54]–[56].

In all countries except for the Netherlands, a cancer diagnosis was associated with higher chances of receiving specialist palliative care and, in Belgium and the Netherlands, with an earlier initiation. Hence, although efforts are made to extend palliative care to non-cancer patients, they appear to still be an underserved group, despite having a symptom burden comparable to that of people with advanced cancer [57]. However, the proportion of non-cancer patients receiving palliative care in the countries we studied was still higher than that in the UK, a country with a leading role in palliative care, where the proportion of patients with cancer in palliative care services was estimated to lie between 75% and 90% for the same period [12].

Older patients appear to be underserved with regard to specialist palliative care in Italy and Spain but not in Belgium and the Netherlands. There we did not find significant differences between age groups with regards to receiving palliative care from either GPs or specialist teams, which is contrary to findings from earlier studies [16], [18]. Increased awareness of palliative care needs of older people in Belgium and the Netherlands has possibly contributed to this change. Moreover, our results suggest that GPs may play an important role in the provision of palliative care to older people as we did not find a significant association between the patient’s age and GPs reporting that they delivered palliative care.

Across countries, home death was relatively consistently associated with higher chances of receiving specialist palliative care or GPs reporting to have delivered palliative care. Place of death was not associated with whether patients received specialist palliative care in Italy and Spain. However, one must take into account the possibility that many people who die in hospital in Italy and Spain are transferred there only shortly before death and spend the bigger part of the last phase of life at home [14]. Therefore their place of death might not reflect their longest place of residence.

This study is an important first step towards estimating the number of people receiving palliative care, and its timing in the general population of people who die non-suddenly in four European countries. Given that the number of people living with and dying of serious chronic diseases is rapidly increasing, a pressing challenge for future research will be to obtain a better understanding of the mechanisms through which patient-, care-, and policy-related factors affect people’s access to and a timely start of palliative care. This knowledge can then be used to inform future planning and implementation of targeted measures to extend palliative care options across patient groups, care settings and countries.
